# Standardized Extract of *Bacopa monniera* Attenuates Okadaic Acid Induced Memory Dysfunction in Rats: Effect on Nrf2 Pathway

**DOI:** 10.1155/2013/294501

**Published:** 2013-09-03

**Authors:** Subhash Dwivedi, Rajasekar Nagarajan, Kashif Hanif, Hefazat Husain Siddiqui, Chandishwar Nath, Rakesh Shukla

**Affiliations:** ^1^Divisions of Pharmacology and Toxicology, CSIR-Central Drug Research Institute, Sector 10, Jankipuram Extension, Sitapur Road, Lucknow 226031, India; ^2^Faculty of Pharmacy, Integral University, Lucknow 226026, India

## Abstract

The aim of the present study is to investigate the effect of standardized extract of *Bacopa monnieri* (memory enhancer) and Melatonin (an antioxidant) on nuclear factor erythroid 2 related factor 2 (Nrf2) pathway in Okadaic acid induced memory impaired rats. OKA (200 ng) was administered intracerebroventricularly (ICV) to induce memory impairment in rats. *Bacopa monnieri* (BM-40 and 80 mg/kg) and Melatonin (20 mg/kg) were administered 1 hr before OKA injection and continued daily up to day 13. Memory functions were assessed by Morris water maze test on days 13–15. Rats were sacrificed for biochemical estimations of oxidative stress, neuroinflammation, apoptosis, and molecular studies of Nrf2, HO1, and GCLC expressions in cerebral cortex and hippocampus brain regions. OKA caused a significant memory deficit with oxidative stress, neuroinflammation, and neuronal loss which was concomitant with attenuated expression of Nrf2, HO1, and GCLC. Treatment with BM and Melatonin significantly improved memory dysfunction in OKA rats as shown by decreased latency time and path length. The treatments also restored Nrf2, HO1, and GCLC expressions and decreased oxidative stress, neuroinflammation, and neuronal loss. Thus strengthening the endogenous defense through Nrf2 modulation plays a key role in the protective effect of BM and Melatonin in OKA induced memory impairment in rats.

## 1. Introduction

Oxidative stress and neuroinflammation play pivotal role in pathogenesis of Alzheimer's disease (AD) and results in memory impairment [1]. Nuclear factor erythroid 2 related factor 2 (Nrf2) is a transcription factor involved to combat oxidative stress and neuroinflammation by coordinated expression of important antioxidant and detoxification genes (phase II genes) through a promoter sequence known as the antioxidant response element (ARE) [2, 3]. These phase II genes, including heme oxygenase-1 (HO-1) and glutamate cysteine ligase catalytic subunit (GCLC), work together to strengthen cellular defense and scavenges reactive oxygen/nitrogen species (ROS/RNS) and detoxifies electrophiles [4, 5]. Besides exerting a direct toxic effect on biological macromolecules (lipid, protein), these reactive ROS may initiate the inflammatory response by stimulating various inflammatory-signaling cascades genes like nuclear factor-kappa B (NF-*κ*B) which is a pleiotropic transcription factor, involved in the regulation of proinflammatory cytokines. These proinflammatory cytokines may further cause tissue injury results in structural and functional changes [6, 7].

Oxidative stress and proinflammatory stimulation results in Tau hyperphosphorylation [8]. The progressive accumulation of the hyperphosphorylated tau protein in the form of neurofibrillary tangles (NFTs) has been recognized as a major hallmark of AD pathology [9, 10]. Downregulation of the PP2A activity is responsible for the abnormal tau phosphorylation in AD brain [11, 12]. The level of NFTs in the neocortex of AD patient is positively correlated with the severity of dementia [13]. These pathological aspects of AD, hyperphosphorylated tau protein, and decreased PP2A activity are well characterized in OKA induced memory impairment model [14]. OKA is present in sea food like dinoflagellates (*Halichondria Okadai*), and it had been observed that the population consuming dinoflagellates suffer from memory impairment. OKA is a selective and potent inhibitor of the serine/threonine phosphatases 1 (PP1) and 2A (PP2A) [15]. Recently, it has been demonstrated that central administration of OKA mimics the characteristics feature of neurodegenerative diseases such as memory impairment, oxidative stress, neuroinflammation, and apoptotic cell death in rodents [16–19].

The pivotal role of cellular oxidation has been highlighted in apoptotic and necrotic cell death [20]. It has been suggested that caspase-3 is a crucial effector whose activation leads to switching on the apoptotic cascade [21]. The B-cell lymphoma 2 (Bcl-2) gene family plays an important role in the control of apoptosis. It includes proapoptotic proteins as Bcl-2-associated X protein (Bax) and antiapoptotic protein as Bcl-2. Thus, the Bax/Bcl-2 ratio appears to be a critical threshold parameter for apoptotic event [22, 23].

In current scenario, antioxidants treatment has been used as a beneficial therapeutic approach in various neurodegenerative diseases [24, 25]. It has also been observed that consumption of fruits and vegetables with high antioxidant activity may decrease the risk of memory deficits in Alzheimer's disease [26]. *Bacopa monniera Linn *(BM) belongs to family Scrophulariaceae. The standardized extract of *Bacopa monniera *(enriched bacoposides A and B-55% ± 5%) has been developed by CSIR-CDRI, Lucknow as memory enhancer, and marketed in the name of Memory Sure by Lumen Marketing Company, Chennai, India. Two principle components, bacoside-A and bacoside-B [27, 28], have been reported for their pharmacological effect. They showed antioxidant and anti-inflammatory potential [29, 30]. Various reports suggest that BM acts as an effective neuroprotective agent against oxidative stress associated different animal models [31–34].

Melatonin (Mel) is an endogenous antioxidant, and its metabolites are potent free radical scavengers, indirect antioxidants [35, 36], and an anti-inflammatory agent [37, 38]. Recently, Melatonin showed protection against exogenous *β*-amyloid deposition induced mitochondrial dysfunction and other neurodegenerative disease models [39–41]. Further, earlier publication from this lab also demonstrated that melatonin protects memory impairments in rats [42, 43]. So Melatonin has been used as standard antioxidant in this study.

However, the molecular mechanism of their antioxidant action and Nrf2 mediated protection in memory impairment is not fully elucidated. Therefore, in present study, we have investigated the effect of BM and Melatonin on Nrf2 pathway in OKA induced neurodegeneration and memory impairment in rats.

## 2. Materials and Methods

All biochemicals unless specified were purchased from Sigma-Aldrich, USA. ELISA kits of TNF-*α*, IL-10 (BD biosciences, USA), IL-1*β* (R&D Systems, USA), NF-*κ*B (Imgenex, San Diego, CA, USA), and Caspase 3 activity kit (Biovision, Milpitas, CA, USA) were procured. Reverse transcriptase primers of different gene like PP2A, GSK-3*β*, Tau, Bax, Bcl2, and *β*-actin were obtained from IDT, USA. *Bacopa monnieri *(Memory sure, Lumen Marketing Company, India) and Melatonin (Sigma) were used in present study.

### 2.1. Animals

Experiments were carried out in male Sprague-Dawley rats (220–250 g) and procured from the Division of Laboratory Animal Services, Central Drug Research Institute, Lucknow, India. All experimental protocols were approved by Institutional Animal Ethics Committee (IAEC) no.—1/11/Pharmacol/IAEC dated 3.2.2011 and performed according to internationally followed ethical standards. Each group consists of 6 rats, and they are kept in a polyacrylic cages maintained under standard husbandry conditions (room temperature 23–25°C and humidity 60–65%) with a 12 hr light and dark cycle. Food and water were available *ad libitum, *but food was withdrawn 1 h prior to behavioral study.

### 2.2. Intracerebroventricular (ICV) Injection of Okadaic Acid

Rats were anesthetized with chloral hydrate (300 mg/kg, i.p.). The head was positioned in a stereotactic frame, and a midline sagittal incision was made in the scalp. From dental burr holes were drilled in the skull on both the sides over the lateral ventricles using the stereotaxic coordinates: 0.8 mm posterior to bregma, 1.5 mm lateral to sagittal suture, and 3.6 mm ventral from bregma. OKA was dissolved in freshly prepared and sterilized artificial cerebrospinal fluid (aCSF) and injected ICV (200 ng) in a volume of 10 *μ*L bilaterally [16].

### 2.3. Drug Administration and Experimental Protocol

**Figure 1:**
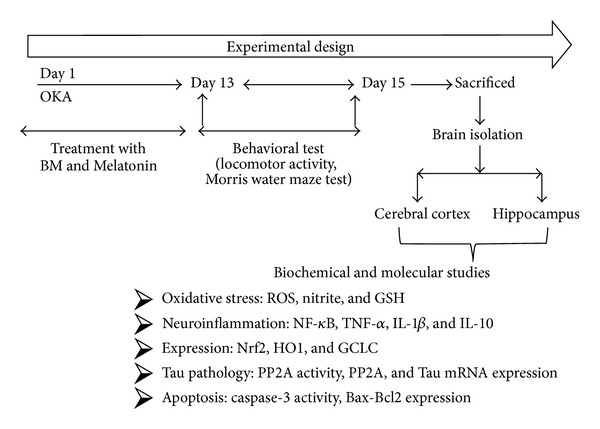


BM (40 and 80 mg/kg) [44] and Melatonin (20 mg/kg) [42] were suspended in 1% (w/v) gum acacia. Drugs were administered orally for 13 days starting from the day of OKA (ICV) injection. Doses were selected and validated for present study on the basis of our earlier lab and others published report (Figure 1).

*Group 1*. Control rats treated with vehicle of BM and Melatonin (1.0% (w/v) gum acacia) for 13 days.

*Group 2*. Rats injected (ICV) with aCSF, (vehicle of OKA) once and treated with vehicle of BM and Melatonin for 13 days.

*Group 3*. Rats injected (ICV) with OKA once and treated with vehicle of BM and Melatonin for 13 days.

*Group 4*. Rats injected (ICV) with OKA once and treated with BM (40 mg/kg) for 13 days.

*Group 5*. Rats injected (ICV) with OKA once and treated with BM (80 mg/kg) for 13 days.

*Group 6*. Rats injected (ICV) with OKA once and treated with Melatonin (20 mg/kg) for 13 days.

*Group 7*. Rats treated with BM (80 mg/kg) for 13 days in *per se.*

*Group 8*. Rats treated with Melatonin (20 mg/kg) for 13 days in *per se.*

### 2.4. Behavioral Studies

#### 2.4.1. Morris Water Maze Test

Morris water maze (MWM) test was used to measure the spatial learning and memory ability of rats [16, 45]. The MWM apparatus consisted of a circular water tank (120 cm diameter, 50 cm high). A platform invisible to the rats was set 2 cm below the water level inside the tank with water maintained at 25 ± 1°C. On 13th day after OKA (ICV) injection, spatial learning and memory of animals were tested in Morris water maze. The rats received 5 consecutive daily training trials in the following days 13, 14, and 15, each trial having a limit of 120 s and a trial interval of approximately 30 s. For each trial, each animal was placed into the water at one of four starting positions, the sequence of which was selected randomly. During trials, rats were placed into the tank at the same starting point, with their heads facing the wall. The animal had to swim until it climbed onto the platform submerged beneath the water. After climbing onto the platform, the animal remained there for 20 s before commencement of the next trial. The escape platform was kept in the same position relative to the distal cues. If the animal failed to reach the escape platform within the maximally allowed time of 120 s, it was guided with the help of a rod and allowed to remain on the platform for 20 s. During a session, latency time to reach the platform and path length was recorded for individual rats and compared by using water maze software (Columbus instrument) in each trial. Mean latency time and path length are calculated. Significant decrease in latency time and path length in subsequent sessions (retention) from 1st session (acquisition) were considered as successful learning.

#### 2.4.2. Probe Trial

A probe trial was performed one hour after the last water maze session at day 15 to access the extent of memory consolidation. The time spent in the target quadrant indicates the degree of memory consolidation that has taken place after learning. The individual rat was placed into the pool as in the training trial, except that the hidden platform was removed from the pool. The time spent in target quadrant was measured for 60 s. In probe trial, each rat was placed at a start position directly opposite to platform quadrant. Further, the path length in target quadrant and number of times crossing over the platform site of each animal were also measured and calculated [46, 47].

#### 2.4.3. Spontaneous Locomotor Activity

OptoVarimex (Columbus Inc., USA) has been used to assess spontaneous locomotor activity (SLA) prior to MWM test to check any change in SLA which may affect the memory test. Every animal was observed for 5 min after an acclimatization period of 10 min. 

### 2.5. Biochemical Estimation

Rats were sacrificed for biochemical estimations by over dose of ether anesthesia and cold saline cardiac perfusion after completion of the behavioral studies. Brains were removed quickly and kept on ice-cold plate and then dissected into cerebral cortex and hippocampus regions [48].

#### 2.5.1. Estimation of GSH

Reduced glutathione (GSH) was determined by its reaction with DTNB to yield a yellow chromophore which was measured spectrophotometrically [49].

#### 2.5.2. Intracellular ROS Estimation

Homogenous suspension of rat brain regions (cerebral cortex and hippocampus) from each group was prepared in HEPES-Tyrode solution (145 mM NaCl, 5 mM KCl, 2 mM CaCl_2_, 1 mM MgCl_2_, 5 mM glucose, 5 mM HEPES, pH 7.4) after treatment with collagenase (750 unit/mL) for 45 min at 37°C. Intracellular ROS was estimated by fluorimetric method using the oxidation sensitive fluorescent probe 2,7 dichlorofluorescein (DCF) diacetate. After collagenase treatment, the dissociated neurons were incubated with DCF (5 *μ*M) for 15 min at 37°C and then washed with HBSS and analyzed by fluorimetry.

#### 2.5.3. Estimation of Nitrite Level

Nitrite level was estimated in brain homogenates by Griess reagent according to previous reports [16, 50].

#### 2.5.4. Serine Threonine Phosphatase II (PP2A) Activity

Lysis buffer with Triton X-100 and protease inhibitors (10 *μ*g/mL Aprotinin, 10 *μ*g/mL Leupeptin, 100 *μ*M PMSF, and 10 *μ*g/mL Pepstatin A) is used for tissue homogenization. Homogenized tissue sample was centrifuge at 10,000 ×g for 10 min at 4°C. Supernatant was collected for the protein phosphatase assay. pNPP (p-Nitrophenyl phosphate) a colorimetric substrate was used for measuring the activity of serine/threonine phosphatases. Upon dephosphorylation by phosphatases, pNPP turns yellow and read at absorbance 405 nm as described earlier [14].

#### 2.5.5. Quantification of NF-*κ*B p65 Unit

The NF-*κ*B/p65 ActivELISA kit (Imgenex, USA) was used to measure NF-*κ*B-free p65 in the nuclear fraction of cerebral cortex and hippocampus. The nuclear levels of p65 may correlate positively with the activation of the NF-*κ*B pathway. The NF-*κ*B ActivELISA is a sandwich ELISA in which free p65 was captured by anti-p65 antibody-coated plates, and the amount of bound p65 was detected by adding a second anti-p65 antibody followed by alkaline phosphatase conjugated secondary antibody using colorimetric detection in an ELISA plate reader at 405 nm. The results were expressed as ng/mg of protein [46].

#### 2.5.6. Proinflammatory and Anti-Inflammatory Cytokines

Rat brain tissues were homogenized in ice-cold Tris buffer (pH 7.2, 4°C) containing 50 mM Tris, 1 mM EDTA, 6 mM MgCl_2_, and 5% (w/v) protease inhibitor cocktail (sigma). After homogenization, samples were sonicated for 10 s using an ultrasonic processor (Heat systems-Ultrasonic, Inc.) at a setting of 20 duty cycle and then centrifuged at 20,800 g for 20 min at 4°C. Supernatants were collected and cytokines were estimated using ELISA kits (R&D Systems and BD biosciences, USA), and readings were taken in Elisa plate reader (Bio-Tek Instruments, Inc.). The quantifications of TNF-*α*, IL-1*β*, and IL-10 were performed by the help and instructions provided by manufacturers. The assay employs the sandwich enzyme immunoassay technique. A monoclonal antibody specific for rat TNF-*α*, IL-1*β*, and IL-10 has been precoated in the microplate. Standards control and samples were pipetted into the wells and any rat TNF-*α*, IL-1*β*, and IL-10 present is bound by the immobilized antibody. After washing away any unbound substance, an enzyme-linked polyclonal antibody specific for rat TNF-*α*, IL-1*β*, and IL-10 was added to the wells. Following a wash to remove any unbound antibody-enzyme reagent, a substrate solution is added to the wells. The enzyme reaction yields a yellow product when the stop solution is added. The intensity of the color measured is in proportion to the amount of rat TNF-*α*, IL-1*β*, and IL-10 bound in the initial steps. Cytokines levels were expressed as pg of cytokines/mg protein [51, 52].

#### 2.5.7. Caspase-3 Colorimetric Assay

Caspase-3, also known as apopain, is an intracellular cysteine protease that exists as a proenzyme and becomes activated during the cascade of events associated with apoptosis. The tissue lysates/homogenates can then be tested for protease activity by the addition of a caspase-specific peptide that is conjugated to the color reporter molecule p-nitroaniline (pNA). The cleavage of the peptide by the caspase releases the chromophore pNA, which can be quantitated spectrophotometrically at a wavelength of 405 nm. The level of caspase enzymatic activity in the cytoplasmic fraction of different brain regions is directly proportional to the color reaction. The results were expressed as percentage of control [17].

### 2.6. Molecular Expression Studies

#### 2.6.1. Reverse Transcriptase PCR (RTPCR)

PP2A, Tau, Bax, and Bcl2 mRNA expressions were studied in cortex and hippocampus of rat brain by reverse transcription polymerase chain reaction. RNA was isolated from brain using TRIzol reagent (Sigma) followed as manufacturer protocol. Concentration and purity of RNA were determined spectrophotometrically using GeneQuant (Amersham Ltd.). Approximately 2 *μ*g of total RNA was reverse transcribed using reverse transcriptase (RT) in a 20 *μ*L mixture containing oligo-(dT)-primer, RNase Inhibitor, dNTP mix, and 5X reaction buffer (Omniscript RT kit) [14].

The resultant cDNA was amplified separately with specific primer for PP2A, Tau, Bax, Bcl2 mRNA, and *β*-actin using Taq PCR core kit (Qiagen, USA). Briefly, cDNA was amplified in a 20 *μ*L reaction volume containing 1 U Taq polymerase, 200 *μ*M (each) dNTP mix, and 2 *μ*L 10X Taq buffer with specific primers. The polymerase chain reaction mixture was amplified in a DNA thermal cycler (Bioer XP cycler) through 35 cycles at the specifications described in Table 1. The PCR products were detected by electrophoresis on a 1.2% agarose gel containing ethidium bromide. Band intensities were quantified by computerized densitometry (Alpha Imager gel documentation system) and normalized with respect to actin mRNA.

### 2.7. Western Blotting

Protein concentration was measured in samples by the method of [53]. Equal proteins from all brain regions were loaded for SDS-PAGE for separation, followed by transfer onto a polyvinyl difluoride (PVDF) membrane. The membrane was kept in blocking 5% BSA containing, Tris-buffer saline (TBS), pH 7.6 for 2 h, and then incubated overnight at 4°C with primary antibodies against Nrf2, HO-1, GCLC, and *β*-Actin (1 : 500 dilution, Santa Cruz Biotechnology, Inc.). Following the incubation, the membrane was washed with washing buffer (pH 7.6, TBS, 0.1% Tween 20) and incubated with secondary antibody conjugated with horseradish peroxidase (1 : 1000 dilution, Santa Cruz Biotechnology, Inc.) for 2 h at room temperature. Following washes with TBS-T, membranes were reacted with ECL reagents (Pierce Biotechnology, Inc.) and exposed to an Image Quant. Relative optical density of protein bands was analyzed using gel documentation system. Protein band variations amounts were normalized to the relative optical density of the *β*-actin band.

### 2.8. Immunohistochemistry

Immunofluorescence technique was performed to investigate the levels of Nrf2 and Phospho-Tau in 12 *μ*m thick cryostat sections of cerebral cortex and hippocampus (coronal section) of rats. The sections were blocked in 10% normal goat serum/0.1 M PBS and then incubated in rabbit Nrf2 (1 : 200. Santa Cruz Biotechnology, Inc) and mouse Phospho-Tau antibody (1 : 200, Santa Cruz Biotechnology, Inc.) at 4°C overnight. After rinsing in PBS, the sections were incubated with Alexa Fluor 594 goat anti-rabbit IgG conjugate (1 : 400 dilution) and Alexa Fluor 488 goat anti-mouse IgG conjugate (1 : 400) for 60 min. Following a further rinse, the sections were counterstained with Hoechst 33258 (0.2 mM) for 5 min and visualized under a fluorescence microscope, after being cover-slipped on Vectashield medium. The images were then imported into Image-J 1.42q (http://rsb.info.nih.gov/ij/, developed by Wayne Rasband, National Institutes of Health, Bethesda, MD, USA) for quantifying cell fluorescence [54]. The fluorescence of Nrf2 and phosphorylated Tau were quantified with the ImageJ software.

### 2.9. Statistical Analysis

Mean ± S.E.M was calculated by using Graph Pad Prism software. Statistical analysis was performed by one-way analysis of variance (ANOVA) followed by Tukey's test where data of more than two groups were compared. Significance of difference between the two groups was determined by using student *t* test.

## 3. Results

### 3.1. Effect of BM and Melatonin on Memory Function in Morris Water Maze Test

#### 3.1.1. Analysis of Latency Time

**Figure 2:**
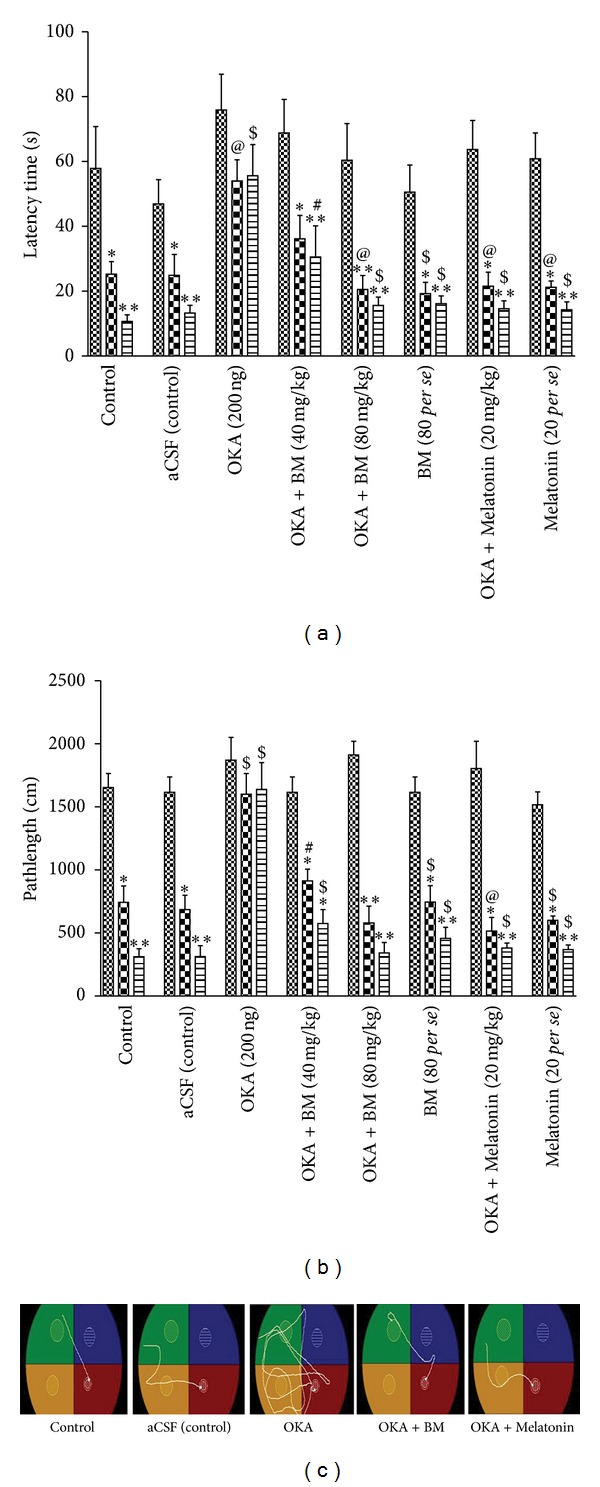


The memory function was assessed in the Morris water maze test. As shown in Figure 2(a) control (*P* < 0.01) and aCSF (control) (*P* < 0.01) groups showed significant decrease in latency time as compared to acquisition trial indicating learning during the retention trials. However, no significant decrease in latency time (*P* > 0.05) was observed in retention trials (sessions 2 and 3) in the OKA treated rats indicating memory impairment. Treatment with BM (40 mg (*P* < 0.05), 80 mg (*P* < 0.001)) and Melatonin (*P* < 0.01) significantly decreased latency time in the OKA treated rats suggesting spatial memory improvement. Intergroup analysis reveals no significant (*P* > 0.05) difference in latency time among the groups in acquisition trial. There was significant difference in latency time in sessions 2 and 3, respectively, as compared with OKA treated animal. Further, there was no significant different in latency time in any session to BM 80 mg and Melatonin 20 mg *per se* treated rats.

#### 3.1.2. Analysis of Path Length

Decrease in path length to search the hidden platform on following days in the water maze test is associated with intact memory in rats. Intragroup analysis showed a significant reduction of path length in control (*P* < 0.01) and aCSF (control) (*P* < 0.01) groups during retention trials as compared to acquisition trial. Further, analysis of path length revealed that OKA treated rats had no significant (*P* > 0.05) reduction in path length as compared with acquisition trial. Treatment with BM (40 mg (*P* < 0.05), 80 mg (*P* < 0.001)) and Melatonin (*P* < 0.001) significantly reduced path length in OKA treated rats, suggesting improvement in memory function (Figure 2(b)). Intergroup analysis reveals no significant (*P* > 0.05) difference in path length among the groups in acquisition trial. There was significant decrease in path length in BM and Mel administered rats during sessions 2 and 3, respectively, as compared with OKA treated animal. Further, there was no significant different in path length in any session to BM 80 mg and Mel 20 mg *per se* treated rats. The improvement in learning and memory is also corroborated by representative swim pattern during last session of trial (Figure 2(c)).

#### 3.1.3. Probe Trial

**Figure 3:**
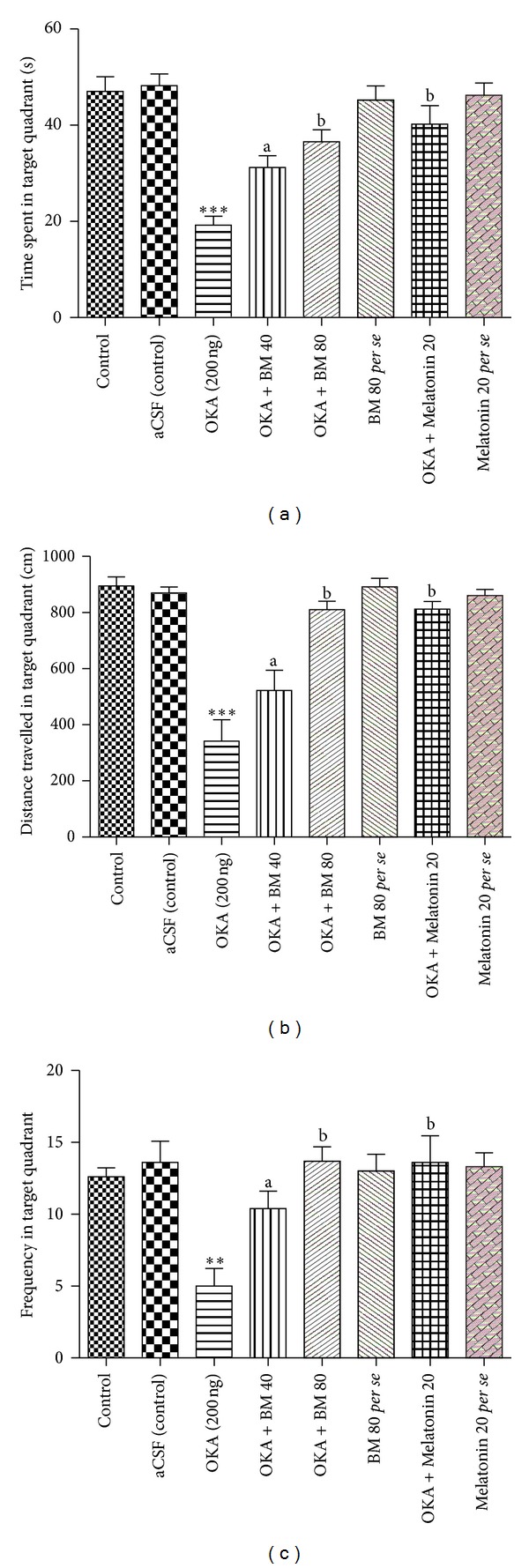


The probe trial data is depicted in Figures 3(a) and 3(c), which provides representations of selective performance in the retention (consolidation) test.  Figure 3(a) is a standard measure and compares time spent in the target quadrant. It was further observed that the target quadrant preference was completely lost in OKA injected rats (*P* < 0.001) in comparison to control and aCSF (control) groups. Treatment with BM (40 mg (*P* < 0.05), 80 mg (*P* < 0.01)) and Melatonin (*P* < 0.01) prevented memory impairment as indicated by the significant (*P* < 0.05) increase in the time spent in target quadrant as compared to OKA group. Further in OKA group, the travelled path length in target quadrant was significantly less (*P* < 0.001) as compared to control and aCSF (control) groups. Treatment with BM and Melatonin prevented the memory impairment as indicated by the significant increase in the path length in target quadrant (*P* < 0.01) as compared to OKA group (Figure 3(b)). Probe trial study also revealed that OKA treated rats showed significantly less (*P* < 0.05) platform crossings when compared with control and aCSF (control) groups, indicating their inadequacy in search accuracy for the hidden platform. BM and Melatonin administration in OKA injected rats improved search accuracy as indicated by significantly higher (*P* < 0.01) platform crossings in comparison to OKA group (Figure 3(c)).

Further, there was no significant different in time spent, distance travelled, and platform crossing in target quadrant in probe trial in BM 80 mg and Mel 20 mg *per se* treated rats.

#### 3.1.4. Effects of Treatment on Locomotor Activity

Table 2 showed that spontaneous locomotor activity did not differ significantly among different groups (*P* > 0.05), ambulatory: (*P* > 0.05) and vertical: (*P* > 0.05).

On the basis of behavioral results, BM 80 mg showed better improvement in memory function in MWM test, and *per se* treatment has no significant effect on memory function as compared with control and aCSF (control) treated rats. Therefore, further biochemical and molecular studies were performed on BM 80 mg/kg and Melatonin 20 mg/kg treated rats.

### 3.2. Biochemical Observations

Effect of BM and Melatonin on ROS, Nitrite, and GSH in OKA induced memory deficit rats.

#### 3.2.1. Effect on Reactive Oxygen Species (ROS)

**Figure 4:**
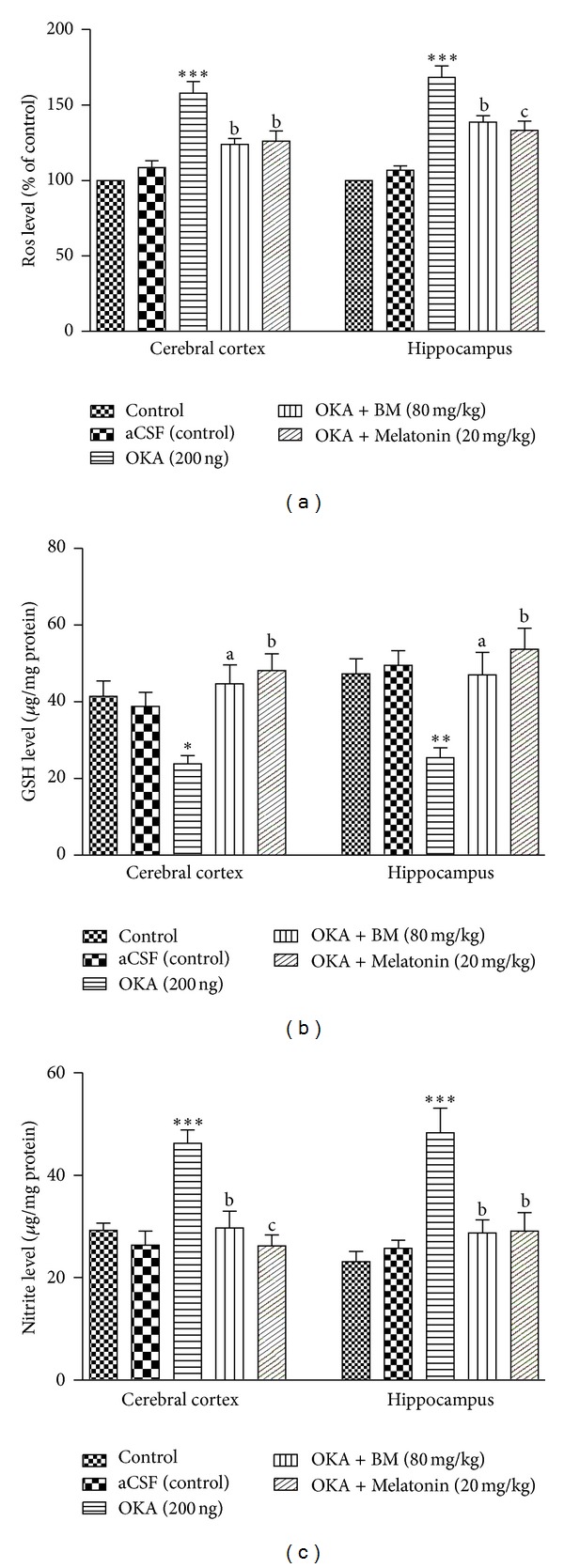


Production of ROS was measured relative to aCSF (control). Treatment with OKA increased ROS generation in cortex (*P* < 0.01) and hippocampus (*P* < 0.01) of rat brain. Treatment with the BM and Melatonin (cortex: (*P* < 0.05) and hippocampus: (*P* < 0.01)) significantly reduced ROS level in both brain parts (Figure 4(a)).

#### 3.2.2. Effect on Glutathione (GSH) Level

As shown in Figure 4(b), administration of the aCSF (control) had no significant (*P* > 0.05) effect on GSH (*μ*g/mg protein) level in any brain region as compared to control. However, a significant fall in the GSH level was observed in cortex (*P* < 0.01) and hippocampus (*P* < 0.01) in OKA treated rats as compared to control and aCSF (control) groups. This reduction in GSH level was ameliorated by preventive treatment with BM and Melatonin (cortex: (*P* < 0.01) and hippocampus: (*P* < 0.01)).

#### 3.2.3. Effect on Nitrite Level

A significant increase in nitrite level was observed in cortex (*P* < 0.001) and hippocampus (*P* < 0.01) of OKA treated rats indicating nitrosative stress. Treatment with the BM and Melatonin (cortex: (*P* < 0.01) and hippocampus: (*P* < 0.01)) caused a significant decrease in nitrite level (Figure 4(c)).

#### 3.2.4. Effect of BM and Melatonin on Neuroinflammation in OKA Induced Memory Deficit Rats

**Figure 5:**
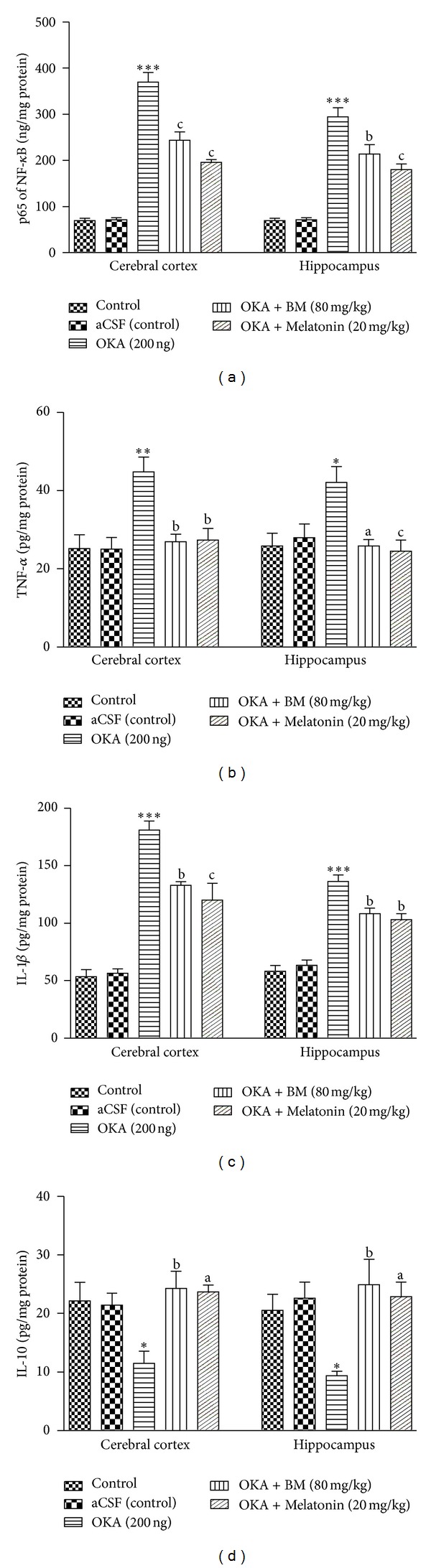


*(1) Effect on NF-*κ*B Activity.* The NF-*κ*B activity was expressed as p65 of NF-*κ*B (ng/mg protein) in the rat brain regions. In comparison to control and aCSF (control) groups, the NF-*κ*B activity elevated significantly in cortex (*P* < 0.01) and hippocampus (*P* < 0.01) of OKA treated rats. Preventive administration of the BM and Melatonin (cortex: (*P* < 0.05) and hippocampus: (*P* < 0.01)) decreased NF-*κ*B activity in brain regions (Figure 5(a)).

*(2) Effect on Tumor Necrosis Factor (TNF-*α*) Level.* As shown in Figure 5(b), the level of TNF-*α* is elevated significantly in cortex (*P* < 0.001) and hippocampus (*P* < 0.001) of OKA treated rats in comparison to control and aCSF (control) groups. Treatment with the BM and Melatonin is restored (cortex: (*P* < 0.05) and hippocampus: (*P* < 0.01)) TNF-*α* level in OKA treated rats.

*(3) Effect on Interleukin-1*β* (IL-1*β*) Level.* Interleukin-1*β* was significantly higher in cortex (*P* < 0.001) and hippocampus (*P* < 0.001) of OKA treated rats in comparison to control and aCSF (control) groups. As shown in Figure 5(c), treatment with the BM and Melatonin (cortex: (*P* < 0.01) and hippocampus: (*P* < 0.01)) is significantly restored the altered IL-1*β* in OKA treated rat brain.

*(4) Effect on Interleukin-10 (IL-10) Level.* As shown in Figure 5(d), administration of the aCSF (control) (ICV) had no significant (*P* > 0.05) effect on IL-10 (pg/mg protein) level in any brain region as compared to control. However, a significant fall in the IL-10 level, as compared to control and aCSF groups, was observed in cortex (*P* < 0.001) and hippocampus (*P* < 0.01) of OKA group. This reduction in IL-10 level ameliorated by preventive treatment with BM and Melatonin (cortex: (*P* < 0.05) and hippocampus: (*P* < 0.01)) significantly restored the altered IL-10 in OKA treated rat brain.

### 3.3. Molecular Observation

#### 3.3.1. Nrf2 Protein Expression

**Figure 6:**
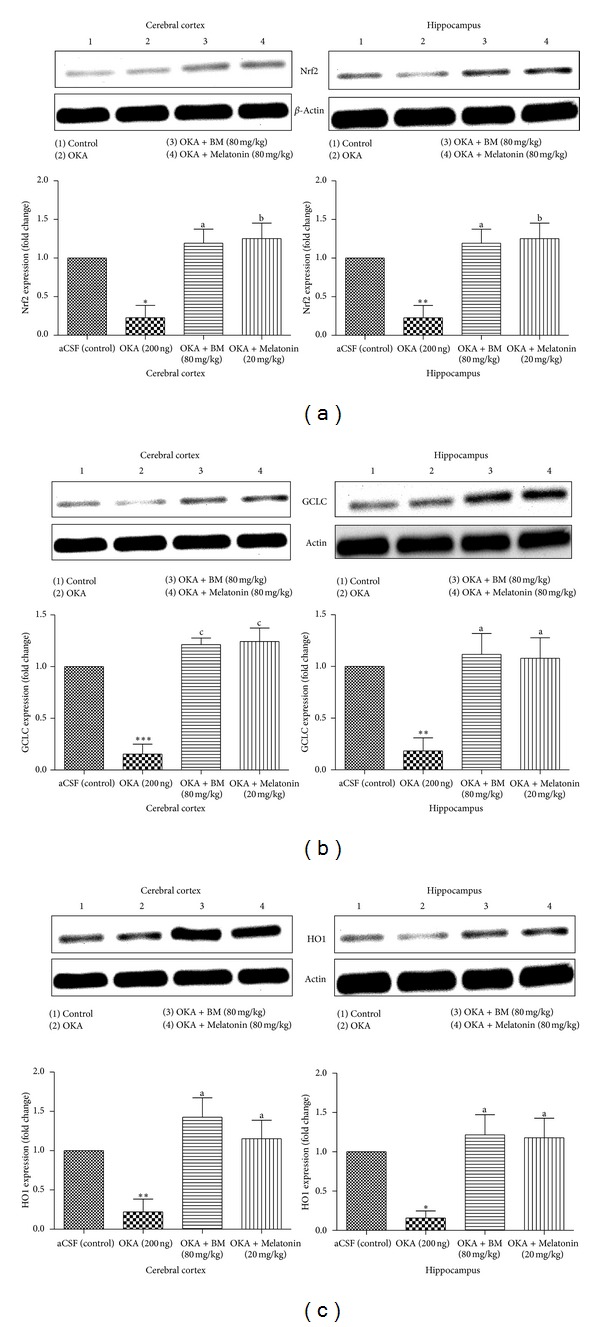


Nrf2 protein expression was studied in cortex and hippocampus. As shown in Figure 6(a), OKA (ICV) caused a significant attenuation in Nrf2 protein level in cortex and hippocampus (*P* < 0.05) in comparison to aCSF (control) group. Chronic administration of BM and Melatonin in OKA injected rat significantly prevented fall in Nrf2 protein expression in cortex (*P* < 0.01) and hippocampus (*P* < 0.05).

#### 3.3.2. HO1 Protein Expression

As shown in Figure 6(b), OKA significantly decreased HO1 protein expression in cortex and hippocampus (*P* < 0.05) in comparison to aCSF (control). This fall in HO1 expression was prevented with BM and Melatonin (cortex: (*P* < 0.01) and hippocampus: (*P* < 0.05)) administration.

#### 3.3.3. GCLC Protein Expression

 OKA (ICV) caused a significant (*P* < 0.05) decrease in GCLC protein expression in cortex and hippocampus in comparison with aCSF (control) group. Chronic treatment with BM and Melatonin in OKA injected rat significantly prevented decrease in GCLC expression in cortex (*P* < 0.01) and hippocampus (*P* < 0.01) regions.

#### 3.3.4. Nrf2 Expression

**Figure 7:**
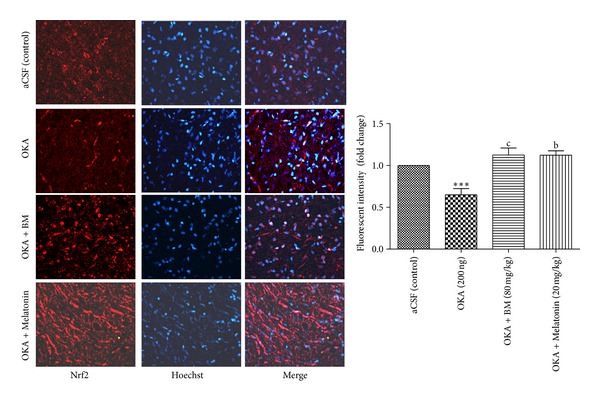


As shown in Figure 7, Immunofluorescent analysis in cortex regions revealed decrease in fluorescent intensity of Nrf2 in OKA induced memory impaired rats. This attenuation in Nrf2 level was significantly (*P* < 0.01) restored by BM and Melatonin administration.

#### 3.3.5. Serine Threonine PP2A Activity

**Figure 8:**
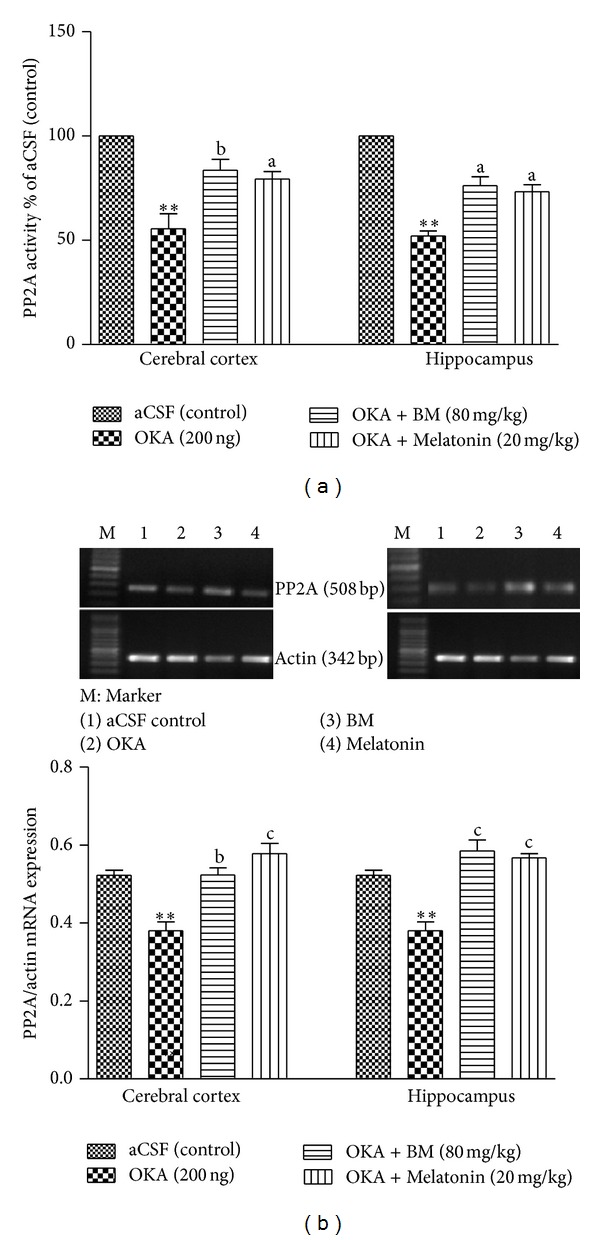


OKA (ICV) significantly (*P* < 0.01) decreased PP2A activity in cortex and hippocampus as compared to control. Whereas treatment with BM and Melatonin significantly (*P* < 0.01) restored PP2A activity in OKA treated cortex (*P* < 0.01) and hippocampus (*P* < 0.01) (Figure 8(a)).

#### 3.3.6. PP2A mRNA Expression

OKA (ICV) significantly (*P* < 0.01) decreased mRNA expression in cortex and hippocampus as compared to control and aCSF groups. Whereas, pretreatment with BM and Melatonin significantly (cortex (*P* < 0.01) and hippocampus (*P* < 0.01)) restored PP2A mRNA expression in OKA treated rats (Figure 8(b)).

#### 3.3.7. GSK3*β* mRNA Expression

**Figure 9:**
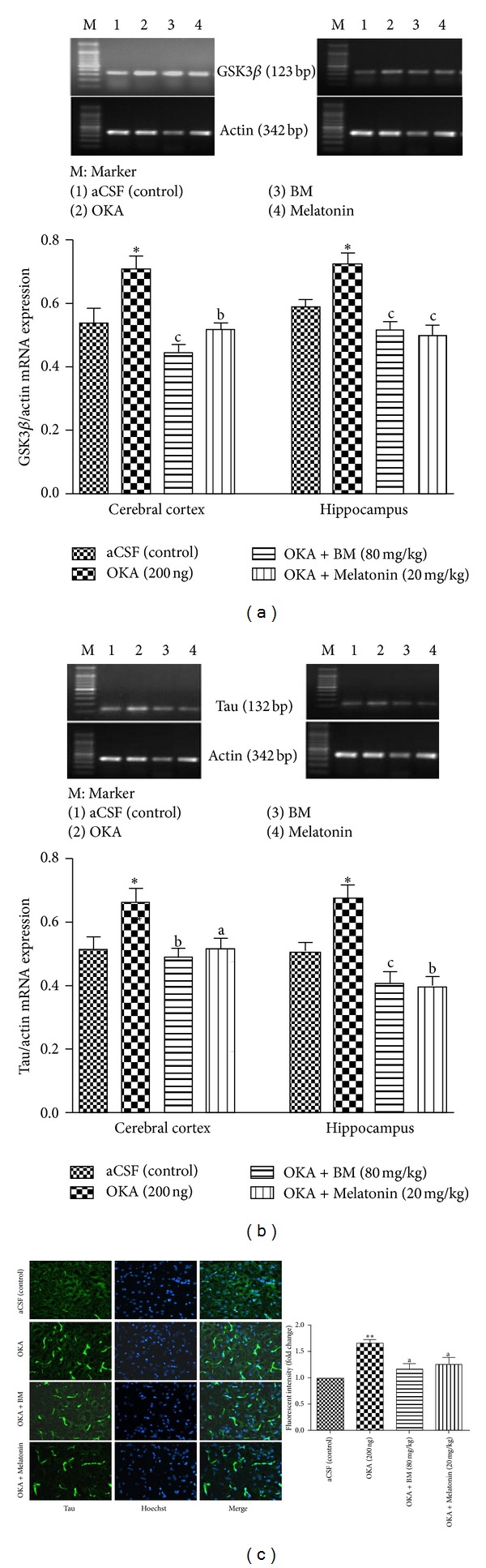


As shown in Figure 9(a), OKA (ICV) caused a significant (*P* < 0.05) increase in GSK3*β* mRNA level in cortex and hippocampus in comparison to aCSF (control) group. Chronic treatment with BM and Melatonin in OKA injected rat significantly prevented decrease in GSK3*β* mRNA expression in cortex (*P* < 0.01) and hippocampus (*P* < 0.05).

#### 3.3.8. Tau mRNA Expression

OKA (ICV) significantly (*P* < 0.05) increased Tau mRNA expression in cortex and hippocampus as compared to aCSF (control) group. (Figure 9(b)). Chronic treatment with BM and Melatonin in OKA injected rat significantly prevented increase in Tau mRNA expression in cortex (*P* < 0.01) and hippocampus (*P* < 0.01) regions.

#### 3.3.9. Tau Expression

Immunofluorescent (Figure 9(c)) analysis of cortex region reveals increase in fluorescent intensity of phosphorylated Tau in OKA induced memory impaired rats. This elevation in Tau expression level was restored (*P* < 0.05) by BM and Melatonin administration.

#### 3.3.10. Bax/Bcl2 Expression and Ratio

**Figure 10:**
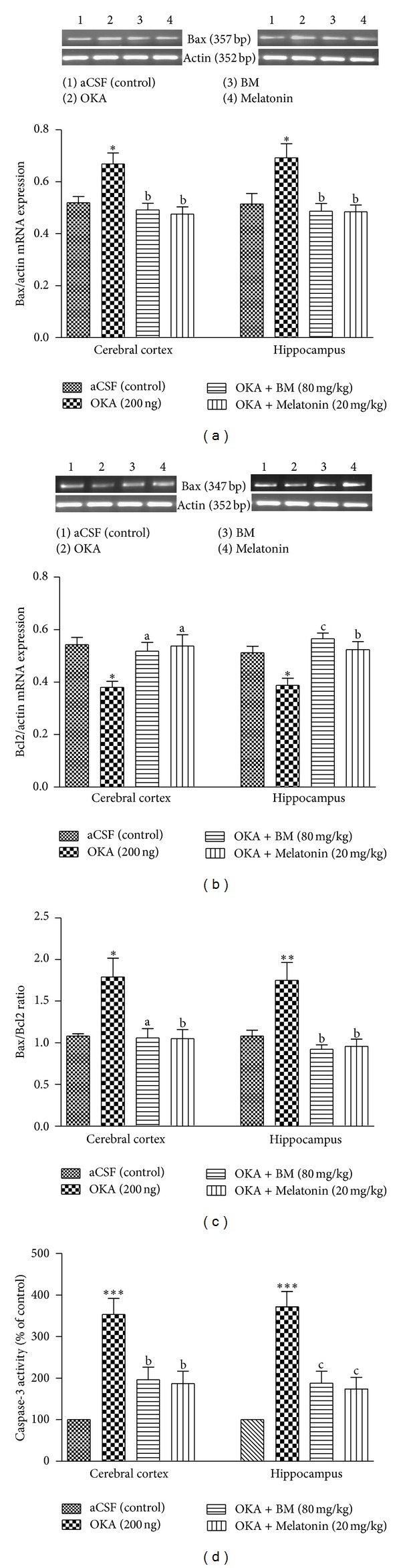


OKA (ICV) significantly (*P* < 0.05) increased Bax (Figure 10(a)) and decreased Bcl2 mRNA (Figure 10(b)) expression in cortex and hippocampus as compared to aCSF (control) group. Chronic treatment with BM and Melatonin in OKA injected rat significantly prevented elevation in Bax and fall in Bcl2 mRNA expression in cortex (*P* < 0.01) and hippocampus (*P* < 0.01). There was significant (*P* < 0.01) elevation in Bax/Bcl2 ratio in OKA treated rats as compared to aCSF (control) group. Administration of BM and Melatonin in OKA injected rat significantly decreased Bax/Bcl2 ratio in cortex (*P* < 0.01) and hippocampus (*P* < 0.01) regions.

#### 3.3.11. Caspase-3 Activity

As shown in Figure 10(d), Caspase-3 activity ((% of aCSF (control)) was significantly (*P* < 0.001) higher in cortex and hippocampus of OKA treated rats in comparison to aCSF (control). Preventive treatment with the BM and Melatonin (cortex: (*P* < 0.05) and hippocampus: (*P* < 0.01)) regions significantly restored the altered Caspase-3 activity in OKA treated rat brain.

## 4. Discussion

The present study was planned to examine the Nrf2 mediated effect of BM and Melatonin on memory function, oxidative stress, neuroinflammation, and neuronal loss in OKA (ICV) induced memory impairment in rats. OKA administration caused impairment in memory function as shown by no significant change in latency time and path length in water maze test. Administration of BM and Melatonin improved memory in OKA injected rats as evidenced by significant reduction in latency time and path length. Further probe trial study also corroborated the memory consolidation and increased target preference in form of time spent in target quadrant, distance travelled, and platform crossing in target quadrant. The locomotor activity was tested by OptoVarimex activity meter, and there was no significant difference among groups excluding the possibility of alteration in locomotors activity which may be contributed in performance of animals in memory tests. Our findings are corroborating earlier reports [42, 44].

OKA administration mimics the characteristics feature associated with neurodegeneration such as memory impairment, oxidative stress, and neuroinflammation [16–18]. Oxidative stress plays imperative role in various neurodegenerative diseases [55, 56]. Activation of endogenous antioxidant enzymes to combat ROS-mediated damage is novel strategy in current scenario. Nrf2, redox regulator, controls the transcription of battery of antioxidant proteins such as HO-1, GCLC, NADPH: quinone oxidoreductase-1 (NQO-1), and glutathione-s-transferases [57]. HO1 acts as antioxidant and anti-inflammatory, while GCLC is rate limiting enzymes in GSH synthesis. OKA administration led to a reduction in Nrf2 level and subsequent lowering in its associated downstream genes HO-1 and GCLC. These results are in accordance with earlier findings in different animal models [57, 58]. Decreased HO-1 and GCLC corroborates increased oxidative stress (decreased GSH and elevated ROS and nitrite level) in OKA treated animal. BM and Melatonin treatment led to significant increase in the Nrf2 level along with its downstream proteins expression and decreased oxidative stress (ROS and nitrite level) and strengthen endogenous antioxidant (GSH level). The restoration of Nrf2 by BM and Melatonin corroborated earlier reports [32, 57].

ROS under oxidative stress may initiate and exaggerate the inflammatory response due to their capability to stimulate and regulate the inflammatory-signaling cascades genes like NF-*κ*B and proinflammatory cytokines [6]. OKA treated rats also showed increased NF-*κ*B activity, proinflammatory cytokines (TNF*α*, IL-1*β*) and attenuated (IL-10 level), this neuroinflammatory status might be due to increased oxidative stress by OKA administration. BM and Melatonin treatment led to significant increase in the Nrf2 level as well as downregulates NF-*κ*B activity and its downstream proinflammatory cytokines (TNF*α*, IL-1*β*). Decreased neuroinflammatory status due to BM treatment further strengthen [59] finding where long-term bacoposides treatment decreases neuroinflammation in aged rats. This decrease in oxidative stress and neuroinflammation with Melatonin treatment may be due to increase in Nrf2/HO1 associated antioxidant enzymes, and inhibition of NF-*κ*B expression also showed in Streptozotocin induced diabetic neuropathy and other models [57, 58].

The balance between the phosphorylation and dephosphorylation of tau is regulated by many kinds of proteinases, such as protein phosphatase-2A (PP2A) and glycogen synthase kinase 3 (GSK-3*β*) is essential. It had been demonstrated that endogenous PP2A is specifically and reversibly inhibited during oxidative stress [60]. Further, the autopsy studies in AD patients also showed the loss of PP2A mRNA, protein, and enzymatic activity in hippocampus and cortex, but not in the cerebellum areas of the brain [61]. Oxidative stress has also been related to GSK-3**β** activation. For example, oxidative stress induces overactivation of GSK-3**β**in neuronal cells [62]. In this study, a significantly decreased PP2A activity/mRNA expression and increased GSK3*β* and Tau mRNA expression in cerebral cortex and hippocampus regions of OKA treated rats have been obtained. As discussed earlier, OKA treated rats showed decreased Nrf2, HO1, and GCLC expression results in oxidative stress which may lead to decreased PP2A and increased GSK3*β*/Tau mRNA expression.

Treatment with BM and Melatonin restored PP2A activity/mRNA expression, decreased GSK3*β* expression, and finally decreased Tau hyperphosphorylation that may prevent NFTs formation and deposition. The restored PP2A activity/expression by Melatonin is corroborating earlier reports where Melatonin attenuates decrease of protein phosphatase 2A in ischemic brain injury and glutamate toxicity induced lowering of PP2A in neuronal cells [63]. Our findings are supporting recent finding [64] where ginsenoside Rb1 is isolated from Ginseng attenuate aluminum induced tau hyperphosphorylation in mice through restoring the GSK-3*β* and PP2A activity. The decreased oxidative stress due to elevation of Nrf2 by these agents may be playing essential role in protection against PP2A/GSK3*β* changes and finally decreased Tau phosphorylation and deposition in OKA induced memory impaired rats.

Under this oxidative stress, neuroinflammation, and Tau phosphorylation environment, excessive free radical attacked membrane phospholipids resulting in mitochondrial membrane potential loss and release of apoptosis-inducing factors that activate apoptosis cascades [65]. Recently involvement of Nrf2 and HO1 pathway in paraquet induced cell damage has been shown [66]. In this study, Okadaic acid induced apoptotic neuronal cell death by decreasing antiapoptotic signaling (Bcl-2) and increasing apoptotic signaling (Bax and caspase-3 activity). Further, [67] has showed that Okadaic acid induced apoptosis in cultured neuronal cells may be due to increase in oxidative stress. Preventive treatment with BM and Melatonin leads to decreased oxidative stress by improving Nrf2 expression and results in improvement in antiapoptotic (Bcl2) expression and decreased proapoptotic (Bax and Caspase-3 activity) indicating neuroprotection. Recently, it has been suggested that Nrf2-mediated transcriptional activation and stabilization of Bcl-2 contribute to reduced apoptosis and promoted cell survival [68]. This finding was supported by [69] where attenuation of apoptotic death in ischemic/reperfusion injury in kidney graft occurred with Nrf2/NF-*κ*B pathway modulation. It has been found that BM protects rat heart against ischaemia-reperfusion injury through modulating the apoptotic regulatory proteins and enzymes (Caspase-3) in line with protection of BM [70] in OKA induced Caspase-3 activation and neuronal apoptosis. Further, It has also been reported that Melatonin decreased beta amyloid induced apoptosis in rat astroglioma C6 cells and improved cell survival [71]. Melatonin administration also decreased Streptozotocin induced neuronal apoptosis (Caspase-3 activity) [43] supporting our results.

In conclusion, the present study demonstrated the activation of Nrf2 and inhibition of NF-*κ*B transcription factors by BM and Melatonin strengthen endogenous defense and protection against OKA induced memory deficit in rats. Therefore, we suggest that Nrf2 might be molecular targets for the development of drugs for memory impairment.

## Figures and Tables

**Table 1:** 

S. no.	Name	Primer sequence	Base pair	Temp (°C)
1	PP2A	Forward 5′-ATG GAC GAG AAG TTG TTC AC-3′	123	55
		Reverse 5′-GAC CAC CAT GTA GAC AGA AG-3′		
2	TAU	Forward 5′-AAG ACA GAC CAT GGA GCA ATC-3′	132	60
		Reverse 5′-CGG CTA ACG TGG CAA GTC TAG-3′		
3	GSK-3*β*	Forward 5′-AGC CTA TAT CCA TTC CTT GG-3′	123	60
		Reverse 5′-CCT CGG ACC AGC TGC TTT-3′		
4	Bax	Forward 5′-GAC TAC GAG GCG TCA TCC-3′	357	55
		Reverse 5′-CCG ATG CTC TGC GCT CTG-3′		
5	Bcl2	Forward 5′-TGG CTT TCT CAT CTC CAT CC-3′	347	55
		Reverse 5′-CTC ACT GCC CCA TTA GTG C-3′		
6	*β*-actin	Forward 5′-GCC ATG TAC GTA GCC ATC CA-3′	352	55.7
		Reverse 5′-GAA CCG CTC ATT GCC GAT AG-3′		

**Table 2:** 

Group	Total	Ambulatory	Vertical
Control	1650 ± 61.02	948.5 ± 101.3	1292 ± 289.8
Vehicle	1431 ± 88.67	756 ± 78.8	1763 ± 205.7
OKA	1694 ± 245.1	999.3 ± 205.5	1412 ± 242
OKA + BM 40	1741 ± 197.5	678.5 ± 67.5	1330 ± 245
OKA + BM 80	1556 ± 312.3	756 ± 78.8	1292 ± 289.8
BM 80 *per se *	1342 ± 231.1	867 ± 68.5	1323 ± 145.4
OKA + Mel	1503 ± 89.56	889.3 ± 214.8	1278 ± 267
Mel *per se *	1723 ± 74.34	879.4 ± 123.5	1324 ± 213.5
